# Receipts Acknowledged

**Published:** 1867-07

**Authors:** 


***Receipts Acknowledged from:***

Geo. F. Heideman, $2; Wm. M. Kaul, 2; T. J. Van Vorhis, 2; J. A. Pinson, 2; A. F. Henry, 2; Morgan & Beadles, 2; J. F. Bell, 2; T. A. Barton, 2; J. W. Brooks, 2; Rob't Steele, 2; B. Wilson, 2; F. A. Mundt, 2; B. K. Shurtleff, 2; W. Anderson, 2; R. L. Hill, 2; C. S. Tucker, 2; B. H. Labree, 1; L. G. Armstrong, 2; Wm. Ziepnecht, 2; T. G. Comstock, 2; S. S. Elder, 2; S. B. McGlumphy, 2; Chas. Kerr, 2; A. H. Scott, 1; G. A. Lamb, 4; H. R. W. Wells, 2; J. Crombie, 2; O. S. Walker, 2; R. H. Crowder, 2; H. B. Upton, 2; J. J. Sherman, 2; P. J. Hermon, 2; H. C. Hull, 1; H. C. Clapp, 2; B. B. Tucker, 2; J. V. Frazier 2; J. H. Newland, 5; L. DePuy, 2; W. H. Gifford, 2; R. Moore, 2; R. S. Kelso, 2; W. F. Watson, 2; E. B. Town, 2; P. B. McKay, 2; E. Smith, 2. W. S. Newton, 2; J. B. Hamilton, 2; W. Bates, 2; W. A. Morse, 2; C. J. Lewis, 2; J. J. Kackley, 2; A. B. Ireland, 2; L. L. Wakefield, 2; A. Catron, 2; C. E. Quire, 2; G. W. Schuchard, 2; C. Jud. Gill, 2; W. W. Weller, 2; C. E. Kuester, 2; J. L. Hoover, 2; Dr. Mergler, 2; E. W. Bogan, 2; J. M. Hagey, 2; H. A. Youmans, 2; A. G. McGrew, 2; John A. Ward, 2; F. M. Agnew, 2; W. D. Wheeler, 2; P. I. Mulcane, 2; L. L. Bennett, 2; A. A. Ames, 4; J. F. Spain, 2; R. S. Hallock, 2; S. S. Clayberg. 2; Amos P. Finch, 2; David Cook, 2; F. W. Hance, 2; F. P. Van Valkenburgh, 2; Thos. M. Sime, 2; P. C. McDonald, 2; O. Peak, 2.

